# Modification of the serotonergic systems and phenotypes by gestational micronutrients

**DOI:** 10.1530/JOE-22-0305

**Published:** 2023-04-19

**Authors:** Vicki Chen, Gia V Shelp, Jacob L Schwartz, Niklas D J Aardema, Madison L Bunnell, Clara E Cho

**Affiliations:** 1 Department of Human Health and Nutritional Sciences, University of Guelph, Guelph, Ontario, Canada; 2 Department of Nutrition, Dietetics and Food Sciences, Utah State University, Logan, Utah, United States

**Keywords:** gestational intake, folic acid, choline, serotonin, metabolic phenotypes

## Abstract

Micronutrients consumed in excess or imbalanced amounts during pregnancy may increase the risk of metabolic diseases in offspring, but the mechanisms underlying these effects are unknown. Serotonin (5-hydroxytryptamine, 5-HT), a multifunctional indoleamine in the brain and the gut, may have key roles in regulating metabolism. We investigated the effects of gestational micronutrient intakes on the central and peripheral serotonergic systems as modulators of the offspring's metabolic phenotypes. Pregnant Wistar rats were fed an AIN-93G diet with 1-fold recommended vitamins (RV), high 10-fold multivitamins (HV), high 10-fold folic acid with recommended choline (HFolRC), or high 10-fold folic acid with no choline (HFolNC). Male and female offspring were weaned to a high-fat RV diet for 12 weeks. We assessed the central function using the 5-HT_2C_ receptor agonist, 1-(3-chlorophenyl)piperazine (mCPP), and found that male offspring from the HV- or HFolRC-fed dams were less responsive (*P* < 0.05) whereas female HFolRC offspring were more responsive to mCPP (*P* < 0.01) at 6 weeks post-weaning. Male and female offspring from the HV and HFolNC groups, and male HFolRC offspring had greater food intake (males *P* < 0.001; females *P* < 0.001) and weight gain (males *P* < 0.0001; females *P* < 0.0001), elevated colon 5-HT (males *P* < 0.01; females *P* < 0.001) and fasting glucose concentrations (males *P* < 0.01; females *P* < 0.01), as well as body composition toward obesity (males *P* < 0.01; females *P* < 0.01) at 12 weeks post-weaning. Colon 5-HT was correlated with fasting glucose concentrations (males R^2^=0.78, *P* < 0.0001; females R^2^=0.71, *P* < 0.0001). Overall, the serotonergic systems are sensitive to the composition of gestational micronutrients, with alterations consistent with metabolic disturbances in offspring.

## Introduction

Diets consumed during pregnancy can induce persistent alterations in the regulation of energy balance and influence susceptibility to chronic diseases (McMillen & Robinson 2005). Both over- and under-nutrition are associated with increased risk of obesity, type 2 diabetes, and cardiovascular disease (Martin-Gronert & Ozanne 2006, Taylor & Poston 2007), with extensive past research that has emphasized metabolic consequences of gestational caloric or macronutrient intakes (Armitage *et al.* 2005, Kind *et al.* 2006). However, limited attention has been on the role of micronutrient intakes during pregnancy in programming the long-term phenotypes of offspring.

Concurrent with the increased prevalence of metabolic diseases (Chew *et al.* 2023), there have been increased intakes of vitamins from supplements and fortified foods (Tarasuk & Brassard 2021, Cowan *et al.* 2023). Studies have revealed high proportions of multivitamin supplement users (Gomez *et al.* 2015, Dubois *et al.* 2017), with the majority of pregnant women consuming supplements at levels reaching or exceeding the Recommended Dietary Allowance for several vitamins. Among the vitamins, folic acid has been found in prenatal supplements in an amount equivalent to or higher than the Tolerable Upper Intake Level of 1000 µg folic acid/day (Wilson *et al.* 2015, Dubois *et al.* 2017). With high-dose supplements together with discretionary fortification practices (Valerie 2014), maternal folic acid intakes of 2.5-fold to even 10-fold higher than requirement have been reported (Bailey *et al.* 2019, Moore *et al.* 2020) and the observation of abnormally high blood folate concentrations (Fayyaz *et al.* 2014, Plumptre *et al.* 2015). Concerns have recently been raised about such excess intakes of micronutrients with unknown metabolic ramifications (Lamers *et al.* 2018, Maruvada *et al.* 2020).

We have previously shown that high (10-fold, non-toxic) intakes of multivitamins or folic acid alone during pregnancy lead to male offspring with characteristics of the metabolic syndrome later in life and associated changes in the central and peripheral systems involved in energy regulation (Cho *et al.* 2013*a*,*b*). With emerging evidence indicating that gut microbes have crucial roles in determining health outcomes (Valdes *et al.* 2018), our recent interest was describing the gut microbiota composition and function across gestational diet and sex of the offspring. Our focus on the consequences of excess folic acid was extended to incorporate the role of choline (a bioactive micronutrient) because both folic acid and choline are known to modulate the gut microbiota (Gurwara *et al.* 2019) and participate in the inter-dependent biochemical pathways including one-carbon metabolism (Zeisel 2013). Whereas excess folic acid intakes are commonly observed (Bailey *et al.* 2019, Moore *et al.* 2020), only small proportions of the population meet the Adequate Intake requirement for choline (Lewis *et al.* 2014, Wallace & Fulgoni 2016, 2017, Moore *et al.* 2020), and such imbalances between folic acid and choline may have potential adverse outcomes. We have recently shown that choline acts as a strong modulator of the effects of the high folic acid gestational diet with changes in the gut microbiota composition toward obesity that differed in a sex-dependent manner (Mjaaseth *et al.* 2021). However, the mechanisms underlying differences in the metabolic phenotypes including body composition and glucose concentrations have not been established.

Serotonin (5-hydroxytryptamine, 5-HT) is an indoleamine signaling molecule that mediates diverse central and peripheral functions including intake regulation (van Galen *et al.* 2021), mood and behavior (Young & Leyton 2002), vascular tone (Cote *et al.* 2004), immune system (Baganz & Blakely 2013), and gastrointestinal tract functions (Keszthelyi *et al.* 2009). It is estimated that over 90% of total body 5-HT is synthesized by enterochromaffin cells of the gastrointestinal mucosa, whereas the remaining pool is produced by serotonergic neurons of the brainstem raphe nuclei, with much smaller amounts made from other peripheral tissues (Gershon & Ross 1966). Central and peripheral pools of 5-HT are functionally distinct as circulating 5-HT does not readily cross the blood–brain barrier (Lexchin *et al.* 1977). Central 5-HT has been well-known for its role in energy homeostasis with 5-HT signaling in the brain contributing to appetite suppression (Blundell 1984). On the other hand, gut 5-HT has previously been associated with gut motility (Spencer *et al.* 2011) and intestinal inflammation (Shajib *et al.* 2017), with limited studies that focused on its contribution to the risk of metabolic disorders including obesity and type 2 diabetes (Yabut *et al.* 2019). Recent data using pharmacological inhibition and genetic models revealed that the gut microbiota serves as a regulator glucose homeostasis through peripheral 5-HT (Martin *et al.* 2019), highlighting the importance of gut mechanisms in metabolic pathways. However, it is unknown whether the varied composition of gestational micronutrients disrupts the serotonergic systems impacting metabolic regulation.

As an extension of our previous study, we sought to determine the effects of high or imbalanced intakes of micronutrients during pregnancy on the serotonergic systems of offspring, in relation to their body composition and glucose regulation. Our selection to use the 5-HT_2C_ receptor agonist, 1-(3-chlorophenyl)piperazine (mCPP), was based on our previous work indicating hypothalamic modulation of 5-HT_2C_ receptor expression by folic acid (Cho *et al.* 2013*b*). With an emerging role of peripheral 5-HT as a metabolic regulator, two functionally separate mechanisms in the brain and the gut may exist as determinants of the offspring phenotypes. We hypothesized that long-term consequences in the metabolic health of offspring of dams fed excess or imbalanced intakes of micronutrients arise with central and peripheral serotonergic alterations.

## Materials and methods

### Animals and diets

This study was part of the larger endeavor that investigated the impact of imbalanced micronutrients on the metabolic health of male and female offspring, whereby the focus of this project was on serotonergic disruptions as a potential mechanism underlying phenotypes of offspring. First-time pregnant Wistar rats (*n* = 10–12/group) at 2–4 days of pregnancy (Charles River, Wilmington, MA, USA) were singly housed in ventilated plastic cages with bedding in a 12 h light:12 h darkness cycle and were randomized to receive an isocaloric AIN-93G diet (Reeves 1997) containing either the 1-fold recommended amount of vitamins (RV), high multivitamins (HV; 10-fold recommended multivitamins), high folic acid with recommended choline (HFolRC; 10-fold recommended folic acid and 1-fold recommended choline), or high folic acid with no choline (HFolNC; 10-fold recommended folic acid and no choline) (Supplementary Table 1, see section on supplementary materials given at the end of this article; Research Diets, New Brunswick, NJ, USA). The 10-fold dose was based on our previous studies that provided reproducible outcomes of the obesogenic phenotypes and has been confirmed to be non-toxic and non-teratogenic (Cho *et al.* 2013*a*,*b*, Cho *et al.* 2015). The AIN-93G diet contains 2 mg/kg of folic acid based on optimal growth rate (Reeves *et al.* 1993) or the equivalent 400 µg/day in women, and 20 mg/kg (10-fold the requirement) is equivalent to 4000 µg/day in women, which is a dose that is commonly observed in the current intake patterns of excess folic acid (Bailey *et al.* 2019, Moore *et al.* 2020). The absence of choline in the 10-fold high folic acid gestational diet (HFolNC) was to remove any effect of choline in the background of excess folic acid, and no folate-related neural tube defect pathogenesis has been reported with choline deficiency (Beaudin *et al.* 2012). At birth, litters were culled to 10 pups per dam. All dams were fed the RV diet during lactation. At weaning, one male and one female pup per dam were randomly selected, singly housed and fed a high-fat (60 kcal% fat, from mostly lard) AIN-93G RV diet (D12451; Research Diets) for 12 weeks, with the diet composition (in g/kg) of 239.5 lard, 123.8 maltodextrin, 200 casein, 68 sucrose, 25 soybean oil, 50 cellulose, 10 vitamin mixture, 35 mineral mixture, 3 l-cystine, and 2.5 choline bitartrate. All rats had* ad libitum* access to food and water throughout the study. The experimental procedure was approved by the Utah State University Institutional Animal Care and Use Committee (protocol #10113).

### Acute food intake response to serotonin receptor agonist

It is widely recognized that administration of mCPP induces acute hypophagia (suppression of food intake) (Vickers *et al.* 2000) and provides an important tool to assess the functional role of the serotonergic system in feeding behaviors. At 6 weeks post-weaning, male and female offspring received i.p. injections of either mCPP (2.5 mg/kg) or 0.9% saline following a 12-h overnight fast. After the injections, food intake was measured for 1 h. The injections were administered in a counter-balance order with a 72-h washout period in-between.

### Long-term food intake and body weight

Food intake and body weight measures were recorded weekly throughout the study period from weaning to 12 weeks post-weaning in male and female offspring. Food intake and body weight changes were calculated as the difference between measures at post-weaning week and weaning.

### Fat mass, lean mass, and fat mass:lean mass

At 12 weeks post-weaning, fat mass and lean mass of male and female offspring were scanned using magnetic resonance imaging (MRI) with EchoMRI-700 (EchoMRI, Houston, TX, USA). Body composition was calculated as a ratio of fat mass to lean mass.

### Fasting blood glucose concentrations

At weaning and 12 weeks post-weaning, offspring were terminated by rapid decapitation following an overnight fast. Fasting blood glucose concentrations were measured directly from trunk blood using a glucose meter (Precision Xtra, Abbot Laboratories).

### Colon 5-HT concentrations

Upon termination at 12 weeks post-weaning, the entire length of the colon from offspring was excised, immediately frozen on dry ice and stored at −80°C until further analyses. A distal section of the emptied colon tissue was weighed and homogenized in PBS buffer (0.9% NaCl) supplemented with 0.1% ascorbic acid at 50 mg/mL. The total protein content of the colon tissue lysates was quantified using a bicinchoninic acid assay kit (Pierce, Cambridge, NJ, USA). Colon 5-HT concentrations were measured using enzyme-linked immunosorbent assay (ELISA) according to the manufacturer’s protocol (SEU39-K01, Eagle Biosciences, Amherst, NH, USA) intended for low concentrated samples including tissue homogenates. Readings from tissue samples were normalized to total protein content.

### Statistical analyses

SAS statistical software Version 9.4 (SAS Institute Inc) was used for all data analyses. Normal distribution of datasets was confirmed by testing for normality. A two-way repeated measures analysis of variance (ANOVA) by the PROC MIXED model procedure followed by Tukey’s *post hoc* test was used to determine the effect of gestational diet, injection and diet × injection interaction on 1-h food intake response. When a diet × injection interaction was significant, food intake differences (expressed as food intake response after mCPP − food intake response after saline) were compared among the diet groups using a one-way ANOVA followed by Tukey’s *post hoc* test. Food intake and body weight changes from weaning to 12 weeks post-weaning were analyzed using a repeated measures ANOVA by the PROC MIXED procedure with gestational diet and time as the main factors and a diet × time interaction term, followed by Tukey’s *post hoc* test. To compare the effects of gestational diets on food intake, body weight, fat mass, lean mass, a ratio of fat mass to lean mass, fasting blood glucose concentrations and colon 5-HT concentrations, a one-way ANOVA followed by Tukey’s *post hoc* test was performed. The four-parameter logistic regression was used to calculate colon 5-HT concentrations from their corresponding optical density. Pearson’s correlation analyses were conducted to determine the association between colon 5-HT and fasting blood glucose concentrations at 12 weeks post-weaning. Food intake response to mCPP and body composition measures had a sample size of *n* = 8–11/group due to injection failures or technique errors. All results are expressed as mean ± standard error of means (s.e.m.). *P* ≤ 0.05 was considered to be statistically significant.

## Results

### Food intake response after mCPP injection

**Figure 1:**
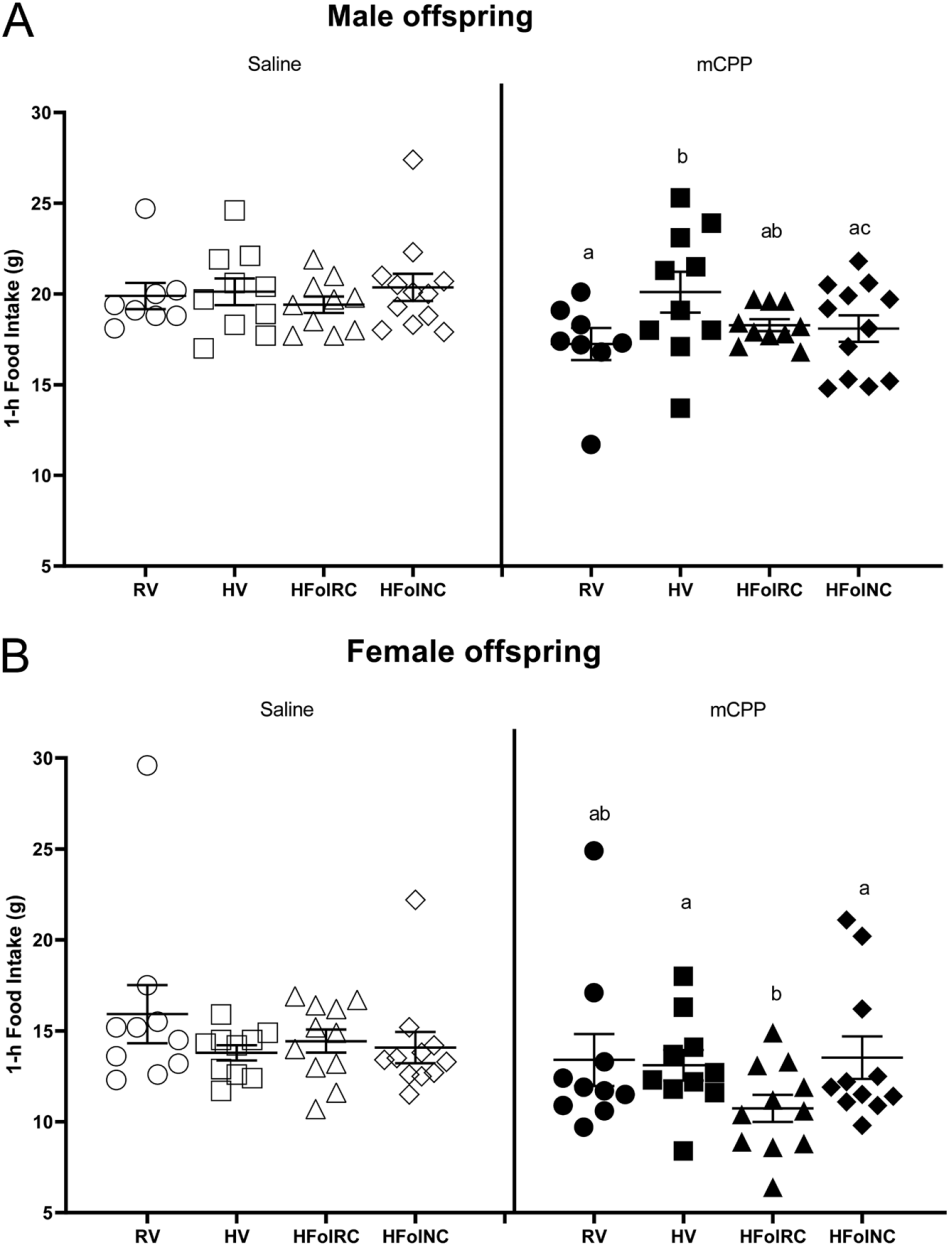
Short-term (1-h) food intake response, in grams, after i.p. injections of 0.9% saline and mCPP (2.5 mg/kg) at 6 weeks post-weaning in (A) male and (B) female offspring from Wistar rat dams fed an AIN-93G diet with either RV: 1-fold recommended vitamins; HV: high 10-fold multivitamins; HFolRC: high 10-fold folic acid with recommended choline; or HFolNC: high 10-fold folic acid with no choline; during pregnancy. (A) Gestational diet *P* = NS, injection *P* < 0.0001, gestational diet × injection *P* < 0.05; (B) gestational diet *P* = not significant, injection *P* < 0.0001, gestational diet × injection *P* < 0.01. Differences in food intake responses (mCPP − saline) were compared among the gestational diet groups. ^ab^*P* < 0.05 by one-way ANOVA followed by Tukey’s *post-hoc* test. NS denotes not significant. Values are mean ± s.e.m..

Following an overnight fast, there was an effect of injection (*P* < 0.0001 in male and female offspring) and gestational diet × injection interaction (*P* < 0.05 in male offspring; *P* < 0.01 in female offspring) without an effect of diet alone on 1-h food intake response at 6 weeks post-weaning. In male offspring, there was a diminished response to mCPP in the HV group compared to the control RV group (*P* < 0.05; Fig. 1A), where the expected food intake suppressive effect was not evident. HFolRC and HFolNC offspring did not differ compared to the RV group in their food intake response after mCPP, but the response in HFolRC offspring also did not differ from that of HV offspring.

In female offspring, the HV and HFolNC groups did not differ in their food intake response after mCPP compared to the control RV group. Female offspring of HFolRC-fed dams also did not differ in their food intake compared to the RV group but the expected food intake suppressive effect in response to mCPP was greater when compared to those from the HV and HFolNC groups (*P* < 0.01; Fig. 1B).

### Food intake change over 12 weeks post-weaning

**Figure 2:**
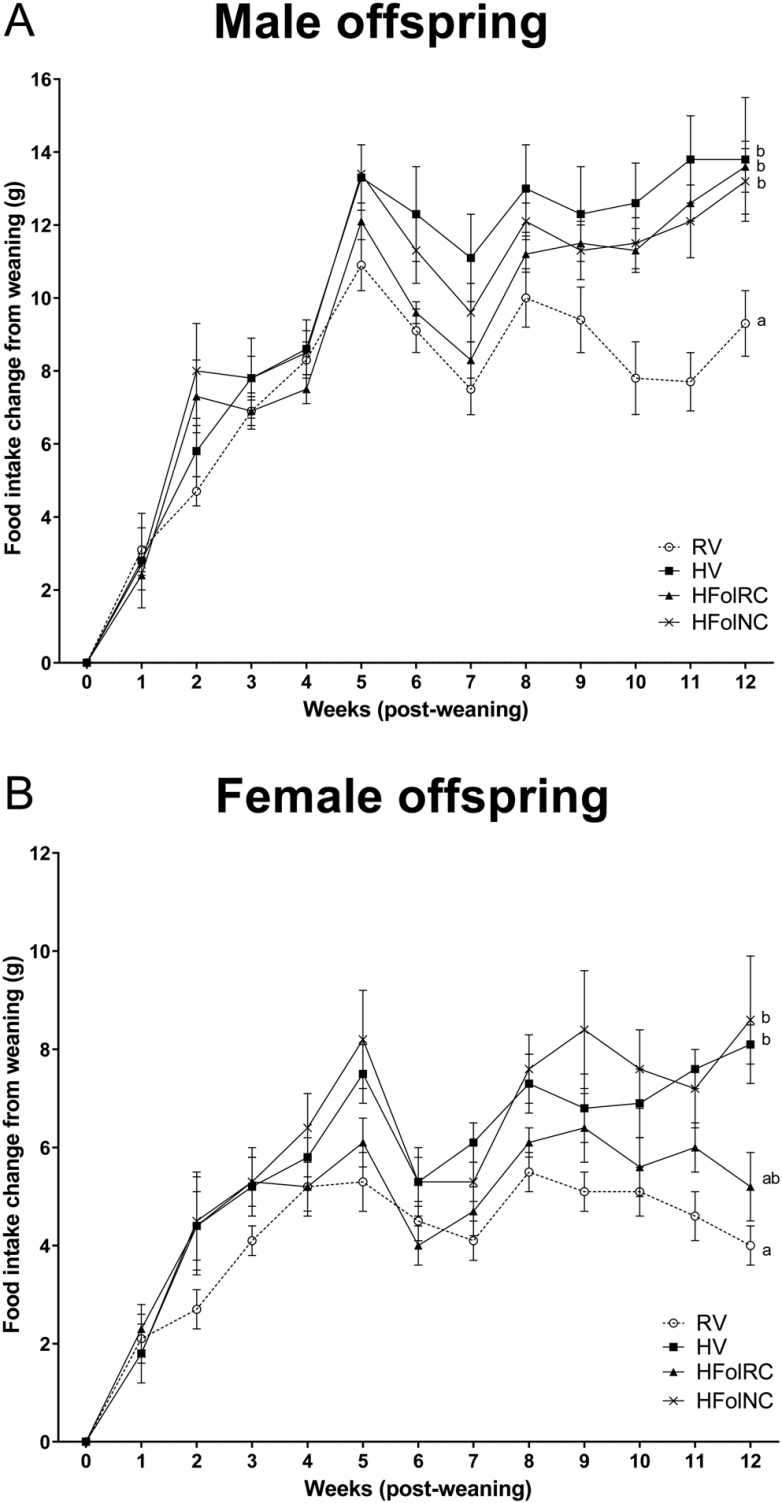
Food intake increase, in grams, from 0-12 weeks post-weaning in (A) male and (B) female offspring from Wistar rat dams fed an AIN-93G diet with either RV: 1-fold recommended vitamins; HV: high 10-fold multivitamins; HFolRC: high 10-fold folic acid with recommended choline; or HFolNC: high 10-fold folic acid with no choline; during pregnancy. (A) Gestational diet *P* < 0.001, time *P* < 0.0001, gestational diet × time *P* < 0.05; (B) gestational diet *P* < 0.001, time *P* < 0.0001, gestational diet × time *P* = not significant. ^ab^ *P* < 0.05 by PROC MIXED model repeated measures ANOVA followed by Tukey’s *post-hoc* test. Values are mean ± s.e.m..

In male offspring, the HV, HFolRC, HFolNC groups had ~25% higher average food intake increase over 12 weeks post-weaning compared to the control offspring (gestational diet *P* < 0.001, time *P* < 0.0001, gestational diet × time *P* < 0.05; Fig. 2A), with no differences among HV, HFolRC and HFolNC offspring. Food intake did not differ among the diet groups at weaning (in grams, RV: 13.7 ± 0.5; HV: 13.5 ± 0.8; HFolRC: 14.0 ± 0.8; HFolNC: 12.8 ± 0.3).

In female offspring, the HV and HFolNC groups had ~40% higher average food intake increase over 12 weeks post-weaning compared to the RV group (gestational diet *P* < 0.001, time *P* < 0.0001, gestational diet × time *P* not significant; Fig. 2B). Food intake change was not different between the HFolRC and RV groups nor among the HV, HFolRC, and HFolNC groups. No differences in food intake among the diet groups were observed at weaning (in grams, RV: 12.5 ± 0.4; HV: 12.7 ± 0.8; HFolRC: 11.3 ± 0.6; HFolNC: 12.2 ± 0.3).

### Body weight gain over 12 weeks post-weaning

**Figure 3:**
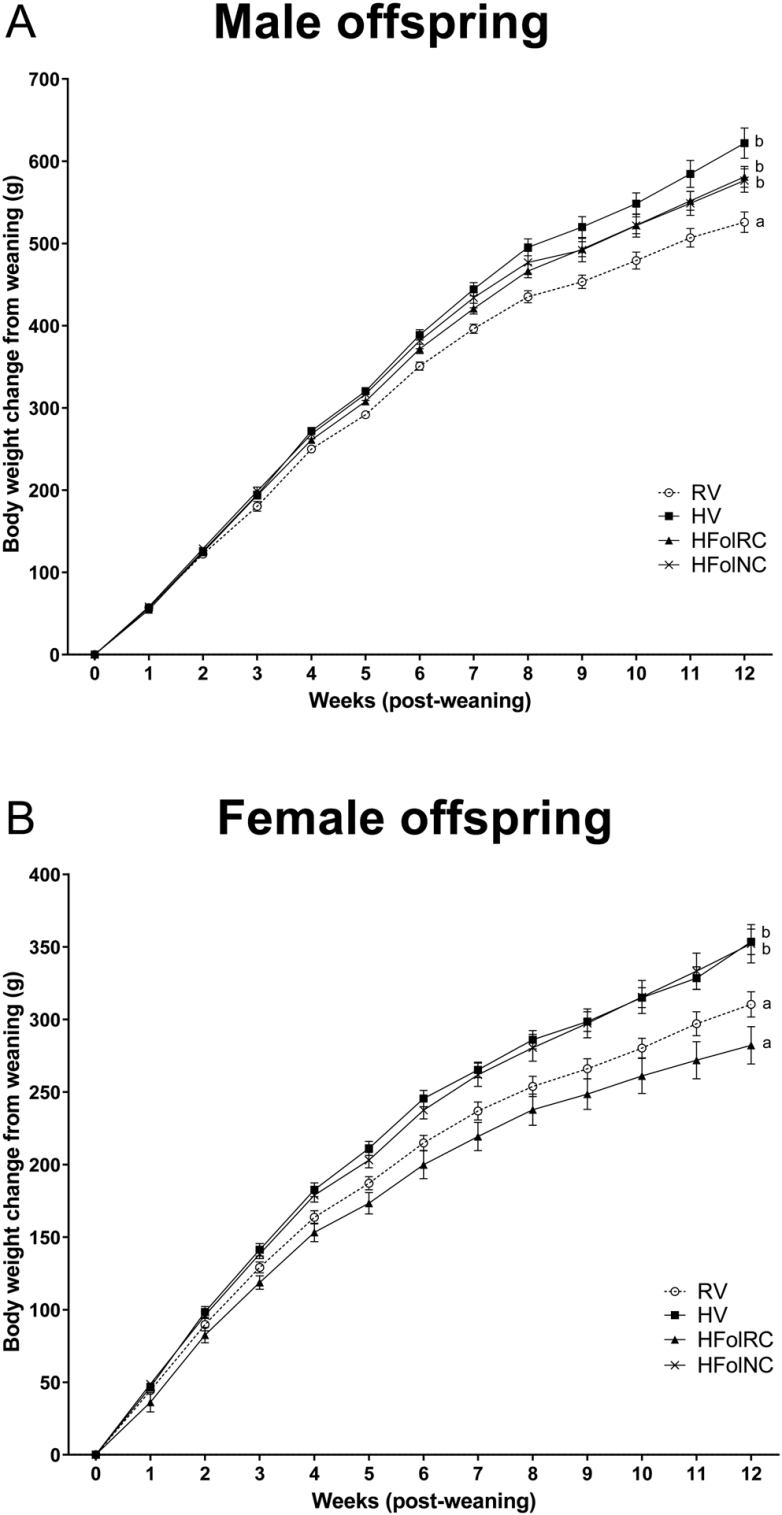
Body weight gain, in grams, from 0 to 12 weeks post-weaning in (A) male and (B) female offspring from Wistar rat dams fed an AIN-93G diet with either RV: 1-fold recommended vitamins; HV: high 10-fold multivitamins; HFolRC: high 10-fold folic acid with recommended choline; or HFolNC: high 10-fold folic acid with no choline; during pregnancy. (A) diet *P* < 0.0001, time *P* < 0.0001, diet × time *P* < 0.0001; (B) diet *P* < 0.0001, time *P* < 0.0001, diet × time *P* < 0.0001. ^ab^*P* < 0.05 by PROC MIXED model repeated measures ANOVA followed by Tukey’s *post-hoc* test. Values are mean ± s.e.m.

In male offspring, the HV, HFolRC, and HFolNC groups had ~10% higher average weight gain over 12 weeks post-weaning compared to the RV group (diet *P* < 0.0001, time *P* < 0.0001, diet × time *P* < 0.0001; Fig. 3A). HV, HFolRC, and HFolNC did not differ in weight gain over time from each other. No differences in body weight among the diet groups were detected at weaning (in grams, RV: 61.0 ± 1.8; HV: 63.0 ± 1.8; HFolRC: 63.9 ± 1.7; HFolNC: 63.6 ± 2.6).

In female offspring, the HV and HFolNC groups had ~12% higher average weight gain over 12 weeks post-weaning compared to the RV group (diet *P* < 0.0001, time *P* < 0.0001, diet × time *P* < 0.0001; Fig. 3B), with no differences between HV and HFolNC. HFolRC females did not differ in weight gain compared to RV females but had ~17% lower body weight gain than HV and HFolNC females. Body weight at weaning did not differ among the diet groups (in grams, RV: 58.5 ± 2.0; HV: 62.5 ± 1.4; HFolRC: 57.0 ± 2.2; HFolNC: 60.5 ± 1.8).

### Fat mass, lean mass, and ratio of fat mass to lean mass

**Figure 4:**
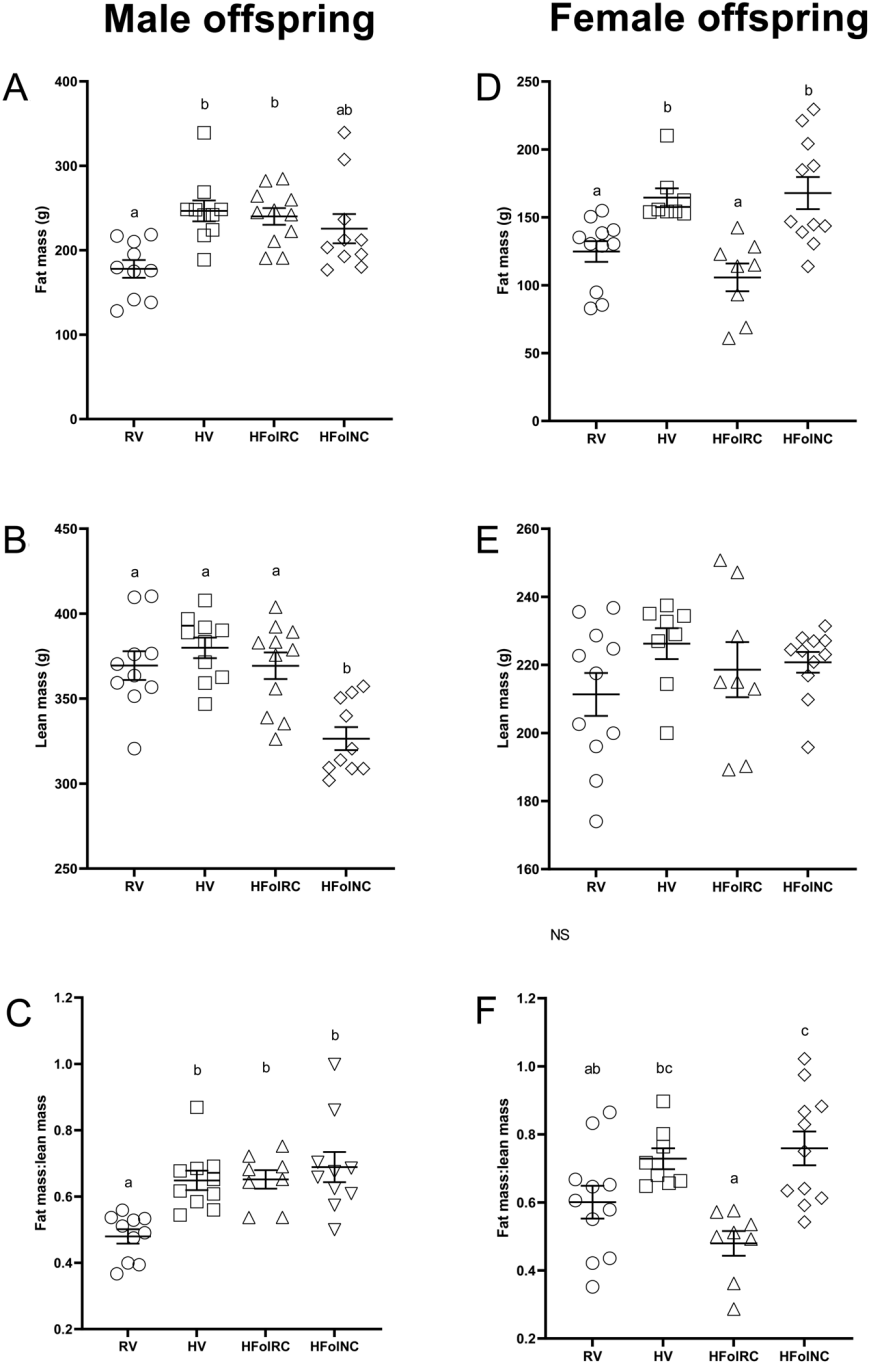
Fat and lean mass, in grams, and fat mass:lean mass ratio at 12 weeks post-weaning, in (A, B, C, respectively) male and (D, E, F, respectively) female offspring from Wistar rat dams fed an AIN-93G diet with either RV: 1-fold recommended vitamins; HV: high 10-fold multivitamins; HFolRC: high 10-fold folic acid with recommended choline; or HFolNC: high 10-fold folic acid with no choline; during pregnancy. ^ab^ *P* < 0.05 by one-way ANOVA followed by Tukey’s *post-hoc* test. Values are mean ± s.e.m.

In male offspring, fat mass was ~37% higher in the HV and HFolRC groups compared to the RV group at 12 weeks post-weaning (*P* < 0.05), with HFolNC offspring that did not differ compared to RV, HV, and HFolRC offspring (Fig. 4A). Lean mass was ~12% lower only in the HFolNC group compared to RV offspring (*P* < 0.0001) with no differences in HV and HFolRC offspring compared to RV offspring (Fig. 4B). Body composition expressed as a ratio of fat mass to lean mass was ~38% higher in HV, HFolRC, and HFolNC offspring compared to RV offspring (*P* < 0.01; Fig. 4C).

In female offspring, the HV and HFolNC groups had ~33% higher fat mass (*P* < 0.001) compared to the RV group, without differences between HFolRC and RV offspring (Fig. 4D). Lean mass did not differ across the diet groups (Fig. 4E), but HFolNC females had ~30% higher ratio of fat mass to lean mass compared to the RV group with differences between HFolRC and HFolNC offspring (*P* < 0.01; Fig. 4F). A ratio of fat mass to lean mass for the HV and HFolRC groups did not differ compared to the RV group.

### Fasting blood glucose concentrations

**Figure 5:**
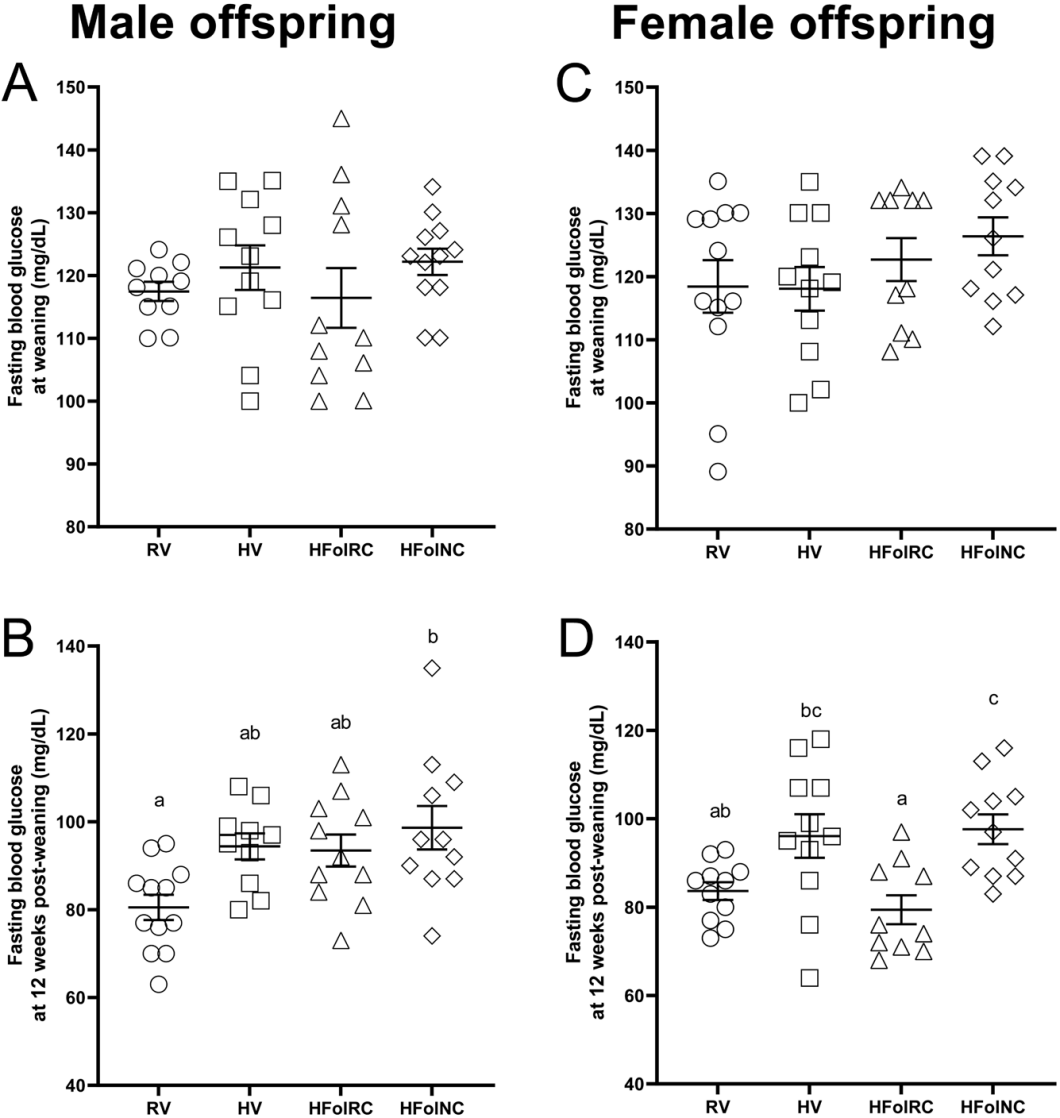
Fasting blood glucose concentrations, in mg/dL, at weaning and 12 weeks post-weaning in (A, B, respectively) male and (C, D, respectively) female offspring from Wistar rat dams fed an AIN-93G diet with either RV: 1-fold recommended vitamins; HV: high 10-fold multivitamins; HFolRC: high 10-fold folic acid with recommended choline; HFolNC: high 10-fold folic acid with no choline; or HFolNC: high 10-fold amount of folic acid with no choline; during pregnancy. ^ab^*P* < 0.05 by one-way ANOVA followed by Tukey’s *post-hoc* test. Values are mean ± s.e.m.

In male offspring, fasting blood glucose was not different among the diet groups at weaning (Fig. 5A), but at 12 weeks post-weaning, ~23% higher levels were observed in HFolNC offspring compared to the RV group (*P* < 0.01; Fig. 5B). HV and HFolRC offspring did not differ in their fasting blood glucose concentrations compared to the RV group, but they also did not differ compared to HFolNC offspring at 12 weeks post-weaning.

In female offspring, fasting blood glucose concentrations also did not differ among the diet groups at weaning (Fig. 5C), but at 12 weeks post-weaning, the HFolNC group had ~17% higher levels compared to the RV group (*P* < 0.01; Fig. 5D). The HV and HFolRC groups did not differ compared to the RV group, but HV offspring were not different from the HFolNC offspring whereas HFolRC offspring had ~18% lower fasting blood glucose than HV and HFolNC offspring at 12 weeks post-weaning.

### Colon 5-HT concentrations

**Figure 6:**
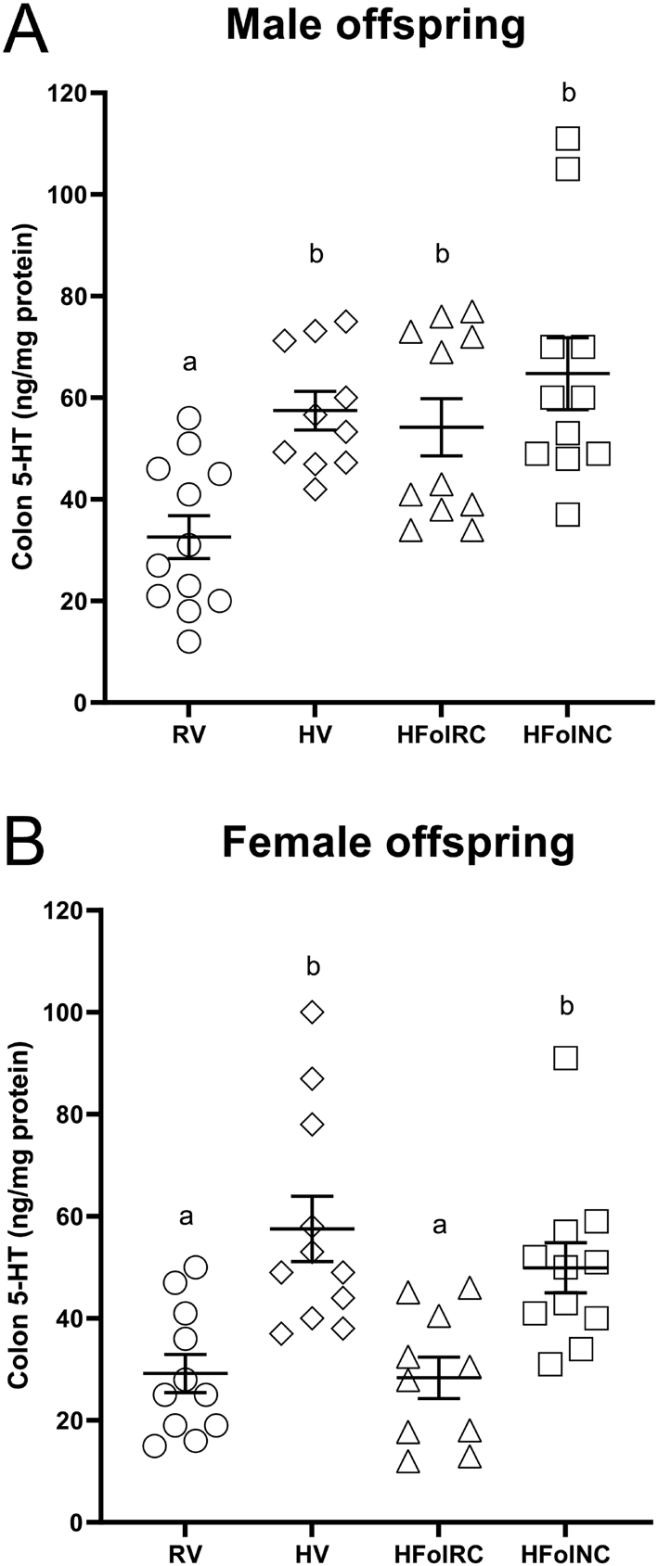
Colon 5-HT concentrations, in ng/mg protein, at 12 weeks post-weaning in (A) male and (B) female offspring from Wistar rat dams fed an AIN-93G diet with either RV: 1-fold recommended vitamins; HV: high 10-fold multivitamins; HFolRC: high 10-fold folic acid with recommended choline; or HFolNC: high 10-fold folic acid with no choline; during pregnancy. ^ab^*P* < 0.05 by one-way ANOVA followed by Tukey’s *post-hoc* test. Values are mean ± s.e.m.

**Figure 7:**
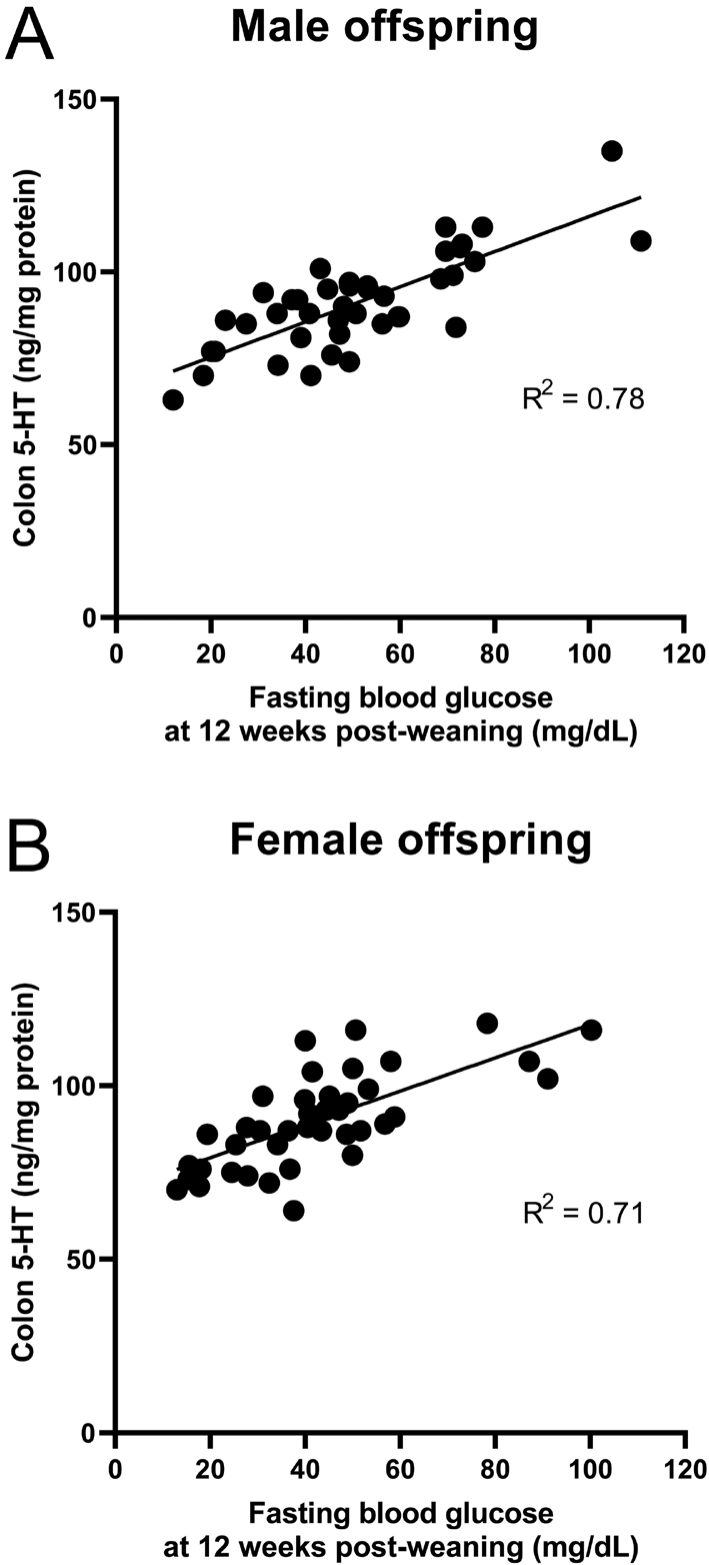
Correlation between colon 5-HT and fasting blood glucose concentrations at 12 weeks post-weaning in (A) male and (B) female offspring from Wistar rat dams fed an AIN-93G diet with either RV: 1-fold recommended vitamins; HV: high 10-fold multivitamins; HFolRC: high 10-fold folic acid with recommended choline; or HFolNC: high 10-fold folic acid with no choline during pregnancy. (A) R^2^ = 0.78, *P* < 0.0001; (B) R^2^ = 0.71, *P* < 0.0001 by Pearson’s correlation analyses.

In male offspring (Fig. 6A), colon 5-HT concentrations were ~80% higher in the HV, HFolRC, and HFolNC groups compared to the RV control at 12 weeks post-weaning (*P* < 0.01). In female offspring (Fig. 6B), the HV and HFolNC groups had ~85% higher colon 5-HT concentrations compared to the RV group at 12 weeks post-weaning (*P* < 0.001). HFolRC offspring did not differ from the RV control but had ~48% lower 5-HT concentrations than HV and HFolNC offspring. Colon 5-HT was positively correlated with fasting blood glucose concentrations in male (R^2^ = 0.78, *P* < 0.0001; Fig. 7A) and female (R^2^ = 0.71, *P* < 0.0001; Fig. 7B) offspring.

## Discussion

Our findings support the hypothesis that excess or imbalanced intakes of micronutrients during pregnancy contribute to alterations of the central and peripheral serotonergic systems, consistent with long-term consequences in the metabolic health of offspring. As 5-HT pools exist in the central and peripheral compartments, we provide compelling evidence that functionally distinct mechanisms may influence metabolic regulation in offspring across diet and sex of the offspring. The central serotonergic effects were evident in the HV and HFolRC groups with disruptions that may explain contrasting phenotypes observed in male vs female offspring from the HFolRC-fed dams. The peripheral serotonergic effects were seen as elevated colon 5-HT levels that matched the obesogenic phenotypes with differences attributed to fat vs lean mass composition. Both male and female offspring from the HFolNC groups displayed high colon 5-HT and fasting blood glucose concentrations with the correlation analyses indicating a strong link between the peripheral serotonergic system and glycemia. These results collectively highlight that the serotonergic pathways are modifiable by gestational nutrition in programming the health trajectory of offspring.

We utilized the 5-HT_2C_ receptor agonist mCPP to assess the central serotonergic function and found acute food intake disruptions that differed by the micronutrient composition in male and female offspring. The expected food intake suppressive response to mCPP was not displayed in male offspring of dams fed the HV or HFolRC diet (with the HV group showing the least responsiveness), suggesting a disruption in the central serotonergic system that may be attributed to folic acid but also other vitamin factors. These results are consistent with the previous study that examined macronutrient selection where protein energy intake was lower in offspring from the control dams but not from HV-fed dams (Szeto *et al.* 2010). Our observed alterations were consistent with long-term food intake and body weight changes from weaning to 12 weeks post-weaning, body composition and glucose concentrations representative of the obesogenic phenotypes, as well as our previous evidence of unregulated energy balance (Mjaaseth *et al.* 2021). Male offspring of dams fed a diet devoid of choline but high in folic acid were not different from the RV group in their response to mCPP, even though the obesogenic phenotypes were evident, suggesting that consequences of an imbalance created between folic acid and choline may not contribute to central 5-HT effects. In female offspring, only those from dams fed a diet high in folic acid with the recommended choline content displayed higher responsiveness to mCPP, consistent with their absence of the obesogenic phenotypes in the current study as well as previous reports (Huot *et al.* 2013, Mjaaseth *et al.* 2021). Differences in food intake response to mCPP across sex of the offspring, where food intake suppression was less in males whereas it was more in females, may explain their contrasting outcomes of the high folic acid gestational diet.

Our observation focusing on the peripheral serotonergic mechanism was novel in showing elevated colon 5-HT in offspring with metabolic disturbances arising from excess or imbalanced micronutrient gestational diets. Colon 5-HT levels were higher in HV, HFolRC, and HFolNC male offspring consistent with the phenotypes of obesity, and our measures of lean and fat mass provided greater insights into consequences impacted by different diets. Male HV and HFolRC offspring had higher fat mass than the control offspring, whereas HFolNC offspring had a fat mass that did not differ from all other groups but had lower lean mass, which HV, HFolRC, and HFolNC all had higher 5-HT concentrations. Similarly, female offspring of HV- or HFolNC-fed dams also had higher 5-HT concentrations consistent with higher fat mass. Colon 5-HT does not appear to distinguish among the different body composition measures as higher fat mass, higher fat:lean mass ratio or reduced lean mass occurred with elevated 5-HT. Previous data indicate that peripheral 5-HT accelerated adipocyte differentiation (Kinoshita *et al.* 2010), highlighting its impact on lipid accumulation and metabolism, and provide support that greater peripheral 5-HT is associated with obesity (Crane *et al.* 2015).

In addition, we showed for the first time that higher colon 5-HT levels from exposure to excess or imbalanced gestational micronutrients occurred concurrently with glucose dysregulation. Male offspring from the HV, HFolRC, and HFolNC groups had higher fasting blood glucose levels, although male HV and HFolRC offspring were not different from the RV or HFolNC group. The strongest effect observed with the HFolNC group may be related to elevated colon 5-HT levels and reduced lean mass, which were distinct from the HV and HFolRC groups that had elevated colon 5-HT levels but also disturbed central serotonergic function and greater fat mass. Thus, body composition toward obesity driven by lower lean mass may influence the relationship between colon 5-HT and hyperglycemia. In female offspring, both the HV and HFolNC groups had higher fasting blood glucose concentrations, although the HV group, similar to males, did not differ from the RV nor HFolNC group. This pattern matched that of fat mass:lean mass ratio, suggesting that both fat and lean mass may be important features associated with glycemia in females. Concentrations of fasting blood glucose and colon 5-HT did not differ between HFolRC and RV females, with more responsiveness to mCPP observed in the HFolRC group, suggesting that folic acid alone in the gestational diet produced distinct effects on the peripheral and central serotonergic systems in female vs male offspring. Higher colon 5-HT levels strongly correlated with fasting blood glucose levels with R^2^ = 0.78 in male and R^2^ = 0.71 in female offspring, and our results align with recent findings that emphasize an important role of the gut serotonergic system as a contributor to metabolic disease risk (Young *et al.* 2015). Overall, these findings raise the possibility that modification of the gut environment involving 5-HT represents a targetable pathway to control host glucose metabolism.

This is the first study to demonstrate that the central and peripheral serotonergic systems are sensitive to the micronutrient composition of the gestational diets with an impact on the metabolic phenotypes of offspring. The supplemental diets of 10-fold reflect a dose that is commonly observed in the western dietary patterns of increased use of vitamin supplements and fortified foods (Bailey *et al.* 2019, Moore *et al.* 2020), thus may have potential application to human settings. However, we note several limitations. First, this study used a folic acid supplemental diet without choline, as our intention was to create a maximal difference between the folic acid and choline amounts, but such an imbalance may not typically occur in humans. Second, a causal relationship cannot be established based on our study design as food intake response to mCPP at 6 weeks post-weaning or concentrations of colon 5-HT at 12 weeks post-weaning in relation to food intake, weight gain, body composition, and blood glucose measures do not indicate which changes occurred first. We are aware that dynamics of food intake and body weight exist, including a rate of adaptation that may be an important predictor of energy regulation (Jacquier *et al.* 2014). Blood glucose concentrations differed at post-weaning, but not at weaning, suggesting that serial measurements across the post-weaning period or intervention studies where the gut microbiota is manipulated in a controlled environment would provide insights into the directionality between 5-HT and phenotypes. Third, we have not identified specific bacterial species responsible for altering colon 5-HT concentrations. Our previous data showed that an imbalance between folic acid and choline led to lower *Bifidobacterium*, *Allobaculum,* and *Lactobacillus vaginalis* toward increased energy harvest (Mjaaseth *et al.* 2021), but further studies are needed to examine specific gut microbial communities as well as gut-derived factors such as short-chain fatty acids (Reigstad *et al.* 2015) as molecular targets in linking colon 5-HT and metabolic health. Fourth, we cannot ignore potential maternal influences on the establishment of the gut microbiota and serotonergic functions, as many studies indicate the role of maternal gut microbiota being transferred to offspring as the basis of programming effects (Walker *et al.* 2017) although other studies distinctly show that diets during pregnancy can independently influence the gut microbiota of offspring (Chu *et al.* 2016). A comparison of the maternal and offspring gut microbiota and functional outcomes will be investigated in our future work. Lastly, it is important to recognize that 5-HT exists in other peripheral sources including adipose tissues (both white and brown adipose tissues) in controlling energy homeostasis (Oh *et al.* 2015). Further studies will be required to decipher the relative contributions of various 5-HT sources as well as different 5-HT actions derived from site-specific vs systemic levels, paying close attention to analytical techniques for different sample types, for example, tissue lysates and platelet-free plasma (Brand & Anderson 2011). In addition, as body composition measures varied among the diet groups with differences in colon 5-HT concentrations, comprehensive lipid profiling would enhance mechanistic understanding of serotonergic regulation of metabolism.

In conclusion, the results from this study indicate that the serotonergic systems respond to the composition of vitamins or methyl nutrients in the gestational diet, with alterations consistent with metabolic outcomes in offspring. As 5-HT has central and peripheral pools with distinct effects on energy homeostasis, metabolic differences observed across diet and sex of the offspring may be attributable to perturbations in the serotonergic systems. Broadly, the identification of novel strategies to reduce disease risk would require integration of various cues stemming from the micronutrient composition, gut microbiota and central pathways involving 5-HT and their regulation of overall metabolic functions.

## Supplementary Material

Supplementary Table 1: Gestational diet composition. AIN-93G diet with RV: 1-fold recommended vitamins; HV: high 10-fold multivitamins; HFolRC: high 10-fold folic acid with recommended choline; or HFolNC: high 10-fold folic acid with no choline.

## Declaration of interest

The authors declare no competing financial interests.

## Funding

This work was supported by the Utah Agricultural Experiment http://dx.doi.org/10.13039/100018206 Station (UTAO+1303), Research Catalyst Program at Utah State University http://dx.doi.org/10.13039/100006630 and College of Biological Science Team Building Program at the University of Guelph http://dx.doi.org/10.13039/100008986. VC was supported by the Undergraduate Research Assistantship Program, University of Guelph http://dx.doi.org/10.13039/100008986. CEC holds a Canadian Institutes of Health Researchhttp://dx.doi.org/10.13039/501100000024 Tier II Canada Research Chair.

## Data availability statement

The data that support the findings of this study are available in the methods and/or supplementary material of this article.

## Author contribution statement

VC contributed to the study design, data collection and statistical analyses, interpreted the data and prepared the manuscript. GVS, JLS, NDJA and MLB contributed to the study design and data collection. CEC conceptualized the study design and contributed to the statistical analyses, data interpretation and manuscript preparation. All authors read and approved the final manuscript.
