# Supplementary material for: Modification of the serotonergic systems and phenotypes by gestational micronutrients
Source: J Endocrinol. 2023 Apr 19;257(2):e220305. doi: 10.1530/JOE-22-0305 (PMC10160553; doi:10.1530/JOE-22-0305)
Supplement: Supplementary Table 1: Gestational diet composition. AIN-93G diet with RV: 1-fold recommended vitamins; HV: high 10-fold multivitamins; HFolRC: high 10-fold folic acid with recommended choline; or HFolNC: high 10-fold folic acid with no choline. [file supplementary_table_1.pdf]

Supplementary Table 1: Gestational diet composition. AIN-93G diet with RV: 1-fold recommended vitamins; HV: high 10-fold multivitamins; HFolRC: high 10-fold folic acid with recommended choline; or HFolNC: high 10-fold folic acid with no choline.

	RV (D10012G)		HV (D18072701)		HFolRC (D18072702)		HFolNC (D18072703)	
	g%	(kcal%)	g%	(kcal%)	g%	(kcal%)	g%	(kcal%)
Protein	20	(20)	20	(20)	20	(20)	20	(20)
Carbohydrate	64	(64)	64	(64)	64	(64)	64	(64)
Fat	7	(16)	7	(16)	7	(16)	7	(16)
	g/kg	(kcal/kg)	g/kg	(kcal/kg)	g/kg	(kcal/kg)	g/kg	(kcal/kg)
Casein	200	(800)	200	(800)	200	(800)	200	(800)
L-cystine	3	(12)	3	(12)	3	(12)	3	(12)
Cornstarch	397	(1590)	397	(1590)	397	(1590)	397	(1590)
Maltodextrin 10	132	(528)	132	(528)	132	(528)	132	(528)
Sucrose	100	(400)	10	(40)	100	(400)	100	(400)
Sucrose from vitamin mix	9.75		97.5		9.75		9.75	
Cellulose, BW200	50	(0)	50	(0)	50	(0)	50	(0)
Soybean oil	70	(630)	70	(630)	70	(630)	70	(630)
t-butylhydroquinone	0.014	(0)	0.014	(0)	0.014	(0)	0.014	(0)
Mineral mix S10022G	35	(0)	35	(0)	35	(0)	35	(0)
Vitamin mix V10037	10	(40)	100	(400)	10	(40)	10	(40)
Folic acid	0.002	(0)	0.02	(0)	0.02	(0)	0.02	(0)
Choline bitartrate	2.5	(0)	2.5	(0)	2.5	(0)	0	(0)
Total	1000	(4000)	1000	(4000)	1000	(4000)	998	(4000)
